# Supplementary material for: Investigating the ‘Bolsonaro effect’ on the spread of the Covid-19 pandemic: An empirical analysis of observational data in Brazil
Source: PLoS One. 2024 Apr 18;19(4):e0288894. doi: 10.1371/journal.pone.0288894 (PMC11025779; doi:10.1371/journal.pone.0288894)
Supplement: S1 Table — Sources: Various media; Authors’ compilation. (DOCX) [file pone.0288894.s001.docx]

***S1 Table. Pandemic denialist statements by President Bolsonaro***

|  | **Death and confirmed cases** | **Date** |
| --- | --- | --- |
| On the pandemic and the virus | "Depois da facada, não vai ser uma gripezinha que vai me derrubar, não ";  **​**"Pelo meu histórico de atleta, caso fosse contaminado pelo vírus, não precisaria me preocupar, nada sentiria ou seria acometido, quando muito, de uma gripezinha ou resfriadinho, como bem disse aquele conhecido médico, daquela conhecida televisão."  “está superdimensionado o poder destruidor desse vírus”  “Muito do que tem ali é muito mais fantasia, a questão do coronavírus, que não é isso tudo que a grande mídia propaga”  “Muitos pegarão isso independente dos cuidados que tomem , mas não podemos entrar numa neurose, como se fosse o fim do mundo”  "E daí? Lamento. Quer que eu faça o quê? Eu sou Messias, mas não faço milagre*​*"  “E agora tem essa *conversinha de segunda onda*”  “Tudo agora é pandemia. Tem que acabar com esse negócio. Lamento os mortos, todos nós vamos morrer um dia. Não adianta fugir disso, fugir da realidade, tem que deixar de ser um país de maricas.”  “Nós temos que enfrentar os nossos problemas, chega de frescura e de mimimi. Vão ficar chorando até quando? Temos de enfrentar os problemas » ; “parece que só se morre de Covid” no Brasil.  “Eu tive a melhor vacina: o vírus...” Sem efeito colateral”... | **03/2020**  **03/2020**  **04/2020**  **11/2020**  **12/2020**  **03/2021**  **12/2021** |
|  | **Non-pharmaceutical measures: Lockdown, social distancing and face masks** |  |
| **Social distancing**  **Face masks**  **Lockdown**  **Face masks**  **Face masks**  **Lockdown**  **Lockdown** | “Muitos pegarão isso independente dos cuidados que tomem" ;  “A vida continua, não tem que ter histeria. Não é porque tem uma aglomeração de pessoas aqui e acolá esporadicamente [que] tem que ser atacado exatamente isso"  *eficácia quase nenhuma*  “Vocês não pararam durante a pandemia. Vocês não entraram na conversinha mole de ‘fica em casa’. Isso é para os fracos.”  "Sabia que o tio estava na praia nadando de máscara? Mergulhei de máscara também, para não pegar Covid nos peixinhos"  “Começam a aparecer os efeitos colaterais das máscaras”  “Tem uns idiotas aí, o 'fique em casa'. Tem alguns idiotas que até hoje ficam em casa"  "Eu tenho o poder de, numa canetada, fazer um lockdown no Brasil todo, mas isso não será feito." | **03/2020**  **10/2020**  **12/2020**  **02/2021**  **04/2021** |
|  | **Pharmaceutical measures: vaccination and other medication** |  |
| **Chloroquine**  **Purchase of vaccine by the Ministry of Health**  **Vaccination**  **Vaccination**  **Vaccination**    **Vaccination**  **Chloroquine**  **Chloroquine**  **Chloroquine**  **Vaccination**  **Vaccination** | “Toma quem quiser, quem não quiser, não toma. Quem é de direita toma cloroquina. Quem é de esquerda toma Tubaína.”  :[“Mandei cancelar, o presidente sou eu, não abro mão da minha autoridade”](https://www1.folha.uol.com.br/equilibrioesaude/2020/10/bolsonaro-fala-em-traicao-e-diz-que-nao-vai-comprar-vacina-chinesa.shtml)  Morte, invalidez, anomalia. Esta é a vacina que o Doria queria obrigar todos os paulistanos a tomá-la"  "O presidente disse que a vacina jamais poderia ser obrigatória. [Mais uma que Jair Bolsonaro ganha](https://www1.folha.uol.com.br/equilibrioesaude/2020/11/mais-uma-que-jair-bolsonaro-ganha-diz-presidente-sobre-suspensao-de-testes-da-coronavac.shtml)."  “[Se tomar e virar um jacaré](https://www1.folha.uol.com.br/equilibrioesaude/2020/12/bolsonaro-volta-a-defender-cloroquina-e-diz-que-ninguem-pode-obrigar-aplicacao-da-vacina-contra-covid.shtml)é problema seu. Se virar um super-homem, se nascer barba em mulher ou homem falar fino, ela [Pfizer] não tem nada com isso”  “Isso é um abuso o que está acontecendo. Uma forma de blindar a Covid é a vitamina D. Então, você pega sol”  “Não há nada comprovado cientificamente sobre essa vacina aí”  « canalha é aquele que é contra o tratamento precoce é não apresenta alternativa. Esse é um canalha »  “Tem idiota que a gente vê nas mídias sociais, na imprensa, né?... Vai comprar vacina. Só se for na casa da sua mãe.”  “Fui acometido de Covid. Procurei não me apavorar. Tomei um medicamento que todo mundo sabe qual foi e no outro dia estava bom."  "Defendi que os médicos brasileiros tivessem autonomia para receitar os remédios, uma decisão que pode ter salvado a vida de muitas pessoas"  "vacinados [contra a Covid] estão desenvolvendo a síndrome da imunodeficiência adquirida [Aids]"  (Ministério da Saúde) : não há demonstração de efetividade da vacina "em estudos controlados e randomizados" nem de segurança "em estudos experimentais e observacionais adequados" | **05/2020**  **10/2020**  **11/2020**  **12/2020**  **01/2021**  **03/2021**  **04/2021**  **05/2021**  **10/2021**  **01/2022** |

Sources: Various media; Authors’ compilation.
