# Supplementary material for: Investigating the ‘Bolsonaro effect’ on the spread of the Covid-19 pandemic: An empirical analysis of observational data in Brazil
Source: PLoS One. 2024 Apr 18;19(4):e0288894. doi: 10.1371/journal.pone.0288894 (PMC11025779; doi:10.1371/journal.pone.0288894)
Supplement: S2 Table — Note: All variables are considered at municipal level. * adjusted at state level considering PNAD-C 2019. (DOCX) [file pone.0288894.s002.docx]

***S2 Table. Data sources and variables***

| **Variables** | **Description** | **Source** | **Nb obs. in data source (approx.)** |
| --- | --- | --- | --- |
| Race (White) | % of whites in the municipality [0,1] | Censo 2010 | Sample (1/10): 20 million |
| Sex (Male) | % of males in the municipality [0,1] | Censo 2010 | Sample (1/10): 20 million |
| Higher education | % of people with higher education in the municipality [0,1] | Censo 2010 | Sample (1/10): 20 million |
| GDP/cap | Gross Domestic Product per capita (log) | IBGE, 2018 | 5,568 municipalities |
| Poverty (AE) | % of beneficiaries of *Auxilio Emergencial* [0,1] | Caixa, 2020 | About 300 million people  (data on 9 months) |
| Age (log) | Age on average (log) | Censo 2010 | Sample (1/10): 20 million |
| Life Expectancy (log) | Life expectancy (log) | FIRJAN, 2018 | 5,568 municipalities |
| Nb. Doctors  (/100,000 h) | Rate: Number of doctors per 100,000 inhabitants | IBGE, Health 2019 | 5,568 municipalities |
| Density (log) | Population size/area size (log) | IBGE, 2019 | 5,568 municipalities |
| Area (Rural) | % of residences in rural areas [0,1] | Censo 2010 | Sample (1/10): 20 million |
| Migration | % of migrants (born in another municipality) [0,1] | Censo 2010 | Sample (1/10): 20 million |
| Commuting | % of people who work outside the municipality [0,1] | Censo 2010 | Sample (1/10): 20 million |
| Overcrowding | Number of people per room | Censo 2010 | Sample (1/10): 20 million |
| Favela | % of residences in slums *(favela*) [0,1] | IBGE, 2019 | 5,568 municipalities |
| Vote for Bolsonaro | % vote for Bolsonaro (2014, 2018, 2022 presidential election) [0,1] | TSE (2014, 2018, 2022) | 5,568 municipalities  * nb rounds * elections |
| Informal worker | % informal workers (Censo2010 adjusted using PNADC_2019) [0,1] | Censo 2010* | Sample (1/10): 20 million |
| Vaccination rate | % people vaccinated (relative to the population of the municipality | Minis. Health /Datasus | About 350 million  (nb of vaccines administered) |
| Mobility | Difference % average mobility per municipality/fev.2020 | Facebook | Between 400,000 to 1,200,000 obs. |

*Note: All variables are considered at municipal level*.

* adjusted at state level considering PNAD-C 2019
