# Supplementary material for: Investigating the ‘Bolsonaro effect’ on the spread of the Covid-19 pandemic: An empirical analysis of observational data in Brazil
Source: PLoS One. 2024 Apr 18;19(4):e0288894. doi: 10.1371/journal.pone.0288894 (PMC11025779; doi:10.1371/journal.pone.0288894)
Supplement: S3 Table — Sources: Ministry of Health, IBGE, TSE; authors’ calculations. * p < 0.10, ** p < 0.05, *** p < 0.01, **** p < 0.001. Note: Negative Binomial (NB) model, except for the last column (Poisson State fixed effect). (DOCX) [file pone.0288894.s003.docx]

**S3 Table. Factors associated with the Covid-19 mortality rate - Detailed results**

**(cumulative data: From February 2020 to December 2022)**

|  | (1) | (2) | (3) | (4) | (5) | (6)  Poisson fixed effect |
| --- | --- | --- | --- | --- | --- | --- |
|  |  |  |  |  |  |  |
| **Vote for Bolsonaro**  **(1^st^ round 2018)** | **1.637^****^** | **1.187^****^** | **0.855^****^** | **0.918^****^** | **0.910^****^** | **0.624^****^** |
|  | (0.000) | (0.000) | (0.000) | (0.000) | (0.000) | (0.000) |
| Poverty level |  |  |  |  |  |  |
| (Auxilio Emergencial beneficiaries) |  | 2.465^****^ | 2.534^****^ | 1.631^****^ | 1.609^****^ | 1.926^****^ |
|  |  | (0.000) | (0.000) | (0.000) | (0.000) | (0.000) |
| Age (log) |  | 1.284^****^ | 2.466^****^ | 2.123^****^ | 2.139^****^ | 2.055^****^ |
|  |  | (0.000) | (0.000) | (0.000) | (0.000) | (0.000) |
| Race (White) |  | -0.103^**^ | 0.126^***^ | 0.00933 | 0.0149 | -0.0607 |
|  |  | (0.023) | (0.008) | (0.847) | (0.760) | (0.666) |
| Sex (Male) |  | -2.249^****^ | -1.955^***^ | -1.745^***^ | -1.726^***^ | -1.206 |
|  |  | (0.000) | (0.001) | (0.004) | (0.004) | (0.161) |
| Education (Higher) |  | 2.529^****^ | 1.146^**^ | 0.916^*^ | 0.938^**^ | -0.0304 |
|  |  | (0.000) | (0.016) | (0.051) | (0.046) | (0.957) |
| GDP/cap (log) |  | 0.168^****^ | 0.145^****^ | 0.106^****^ | 0.106^****^ | 0.0785^****^ |
|  |  | (0.000) | (0.000) | (0.000) | (0.000) | (0.000) |
| Life Expectancy (log) |  | 1.786^****^ | 1.425^****^ | 1.144^****^ | 1.142^****^ | 0.565 |
|  |  | (0.000) | (0.000) | (0.000) | (0.000) | (0.268) |
|  |  |  |  |  |  |  |
| Nb. Doctors (/100K h) |  |  | 0.00998 | -0.0216 | -0.0236 | 0.0861 |
|  |  |  | (0.913) | (0.810) | (0.793) | (0.521) |
| Density (log) |  |  | -0.0297^****^ | -0.0250^****^ | -0.0252^****^ | 0.0127 |
|  |  |  | (0.000) | (0.000) | (0.000) | (0.202) |
| Area (Rural) |  |  | -0.515^****^ | -0.521^****^ | -0.519^****^ | -0.590^****^ |
|  |  |  | (0.000) | (0.000) | (0.000) | (0.000) |
| Migration (Migrant) |  |  | 0.406^****^ | 0.406^****^ | 0.403^****^ | 0.0730 |
|  |  |  | (0.000) | (0.000) | (0.000) | (0.500) |
| Job (Commuting) |  |  | 0.142^**^ | 0.0416 | 0.0497 | 0.0484 |
|  |  |  | (0.049) | (0.563) | (0.493) | (0.596) |
| Dwelling (Overcrowding) |  |  | 0.641^****^ | 0.641^****^ | 0.634^****^ | 0.412^**^ |
|  |  |  | (0.000) | (0.000) | (0.000) | (0.049) |
| Location (Favela) |  |  | 0.467^***^ | 0.572^****^ | 0.570^****^ | 0.258^*^ |
|  |  |  | (0.001) | (0.000) | (0.000) | (0.060) |
| Job (Informal) |  |  | -0.150 | -0.0808 | -0.0823 | -0.164 |
|  |  |  | (0.181) | (0.468) | (0.459) | (0.348) |
|  |  |  |  |  |  |  |
| Vaccine rate (1^st^ dose) |  |  |  | 0.00497^****^ | 0.00630^****^ | 0.00402^**^ |
|  |  |  |  | (0.000) | (0.000) | (0.025) |
| Vaccine rate (2^nd^ dose) |  |  |  |  | -0.00143 | -0.000640 |
|  |  |  |  |  | (0.363) | (0.735) |
|  |  |  |  |  |  |  |
| Constant | 4.865^****^ | -8.170^****^ | -10.20^****^ | -7.771^****^ | -7.826^****^ |  |
|  | (0.000) | (0.000) | (0.000) | (0.000) | (0.000) |  |
|  |  |  |  |  |  |  |
| Lnalpha | -1.496^****^ | -1.725^****^ | -1.797^****^ | -1.820^****^ | -1.820^****^ |  |
|  | (0.000) | (0.000) | (0.000) | (0.000) | (0.000) |  |
| *N* | 5568 | 5340 | 5269 | 5269 | 5269 | 5268 |
| pseudo *R*^2^ | 0.027 | 0.042 | 0.048 | 0.050 | 0.050 |  |
| *AIC* | 67996.4 | 64088.9 | 62828.5 | 62709.1 | 62710.3 | 208860.5 |

*Sources*: Ministry of Health, IBGE, TSE; authors’ calculations.

^*^ *p* < 0.10, ^**^ *p* < 0.05, ^***^ *p* < 0.01, ^****^ *p* < 0.001

*Note*: Negative Binomial (NB) model, except for the last column (Poisson State fixed effect).
