# Supplementary material for: Investigating the ‘Bolsonaro effect’ on the spread of the Covid-19 pandemic: An empirical analysis of observational data in Brazil
Source: PLoS One. 2024 Apr 18;19(4):e0288894. doi: 10.1371/journal.pone.0288894 (PMC11025779; doi:10.1371/journal.pone.0288894)
Supplement: S4 Table — Sources: Ministry of Health, IBGE, TSE, Facebook Movement Range; authors’ calculations. * p < 0.10, ** p < 0.05, *** p < 0.01, **** p < 0.001. Note: OLS model. (DOCX) [file pone.0288894.s004.docx]

**S4 Table. Factors associated with the change in mobility – Detailed results**

**(cumulative data: from February 2020 to October 2021)**

|  | (1) | (2) | (3) | (4) | (5) | (6) | (7) |
| --- | --- | --- | --- | --- | --- | --- | --- |
|  |  |  |  |  |  |  |  |
| **Vote for Bolsonaro**  **(1^st^ round 2018)** | **-0.0510****** | **0.0613****** | **0.0607****** | **0.0585****** | **0.0586****** | **0.0573****** |  |
|  | (0.000) | (0.000) | (0.000) | (0.000) | (0.000) | (0.000) |  |
| Poverty level (AE |  |  |  |  |  |  |  |
| (Auxilio Emergencial benef.) |  | 0.0510 | 0.103*** | 0.0958** | 0.0940** | 0.0829** | 0.0460 |
|  |  | (0.133) | (0.005) | (0.011) | (0.018) | (0.045) | (0.259) |
| Age (log) |  | 0.0298 | -0.137**** | -0.143**** | -0.144**** | -0.133**** | -0.141**** |
|  |  | (0.135) | (0.000) | (0.000) | (0.000) | (0.000) | (0.000) |
| Race (White) |  | -0.0681**** | -0.0569**** | -0.0575**** | -0.0577**** | -0.0561**** | -0.0417**** |
|  |  | (0.000) | (0.000) | (0.000) | (0.000) | (0.000) | (0.000) |
| Sex (Male) |  | 1.564**** | 0.881**** | 0.883**** | 0.883**** | 0.883**** | 0.980**** |
|  |  | (0.000) | (0.000) | (0.000) | (0.000) | (0.000) | (0.000) |
| Education (Higher) |  | -0.500**** | -0.511**** | -0.517**** | -0.518**** | -0.502**** | -0.453**** |
|  |  | (0.000) | (0.000) | (0.000) | (0.000) | (0.000) | (0.000) |
| GDP/cap (log) |  | 0.0034 | -0.0004 | -0.0006 | -0.0007 | -0.0008 | -0.00003 |
|  |  | (0.171) | (0.883) | (0.813) | (0.794) | (0.763) | (0.992) |
| Life Expectancy (log) |  | -0.137** | -0.0206 | -0.0217 | -0.0222 | -0.0252 | 0.0278 |
|  |  | (0.021) | (0.724) | (0.710) | (0.705) | (0.667) | (0.632) |
|  |  |  |  |  |  |  |  |
| Nb. Doctors (/100K h) |  |  | 0.000006 | 0.000005 | 0.000005 | 0.000004 | 0.000004 |
|  |  |  | (0.683) | (0.746) | (0.750) | (0.785) | (0.788) |
| Density (log) |  |  | -0.0091**** | -0.0090**** | -0.0090**** | -0.0091**** | -0.0097**** |
|  |  |  | (0.000) | (0.000) | (0.000) | (0.000) | (0.000) |
| Area (Rural) |  |  | -0.0164* | -0.0147 | -0.0147 | -0.0148 | -0.0216** |
|  |  |  | (0.091) | (0.135) | (0.135) | (0.133) | (0.028) |
| Migration (Migrant) |  |  | -0.01000 | -0.0111 | -0.0111 | -0.0107 | 0.000854 |
|  |  |  | (0.303) | (0.254) | (0.257) | (0.271) | (0.929) |
| Job (Commuting) |  |  | -0.0310** | -0.0318** | -0.0320** | -0.0309** | -0.0276** |
|  |  |  | (0.016) | (0.014) | (0.013) | (0.017) | (0.034) |
| Dwelling (Overcrowding) |  |  | -0.0737**** | -0.0752**** | -0.0750**** | -0.0713**** | -0.0746**** |
|  |  |  | (0.000) | (0.000) | (0.000) | (0.000) | (0.000) |
| Location (Favela) |  |  | -0.113**** | -0.114**** | -0.114**** | -0.114**** | -0.108**** |
|  |  |  | (0.000) | (0.000) | (0.000) | (0.000) | (0.000) |
| Job (Informal) |  |  | -0.0230 | -0.0222 | -0.0220 | -0.0224 | -0.0160 |
|  |  |  | (0.353) | (0.370) | (0.376) | (0.366) | (0.522) |
|  |  |  |  |  |  |  |  |
| Mortality rate |  |  |  | 0.0000139 | 0.0000138 | 0.0000135 | 0.0000243* |
|  |  |  |  | (0.268) | (0.277) | (0.286) | (0.053) |
| Vaccine rate (1^st^ dose) |  |  |  |  | 0.0000155 | 0.000224 | 0.000291 |
|  |  |  |  |  | (0.887) | (0.326) | (0.203) |
| Vaccine rate (2^nd^ dose) |  |  |  |  |  | -0.000266 | -0.000401 |
|  |  |  |  |  |  | (0.297) | (0.117) |
|  |  |  |  |  |  |  |  |
| Constant | -0.040**** | -0.373 | 0.094 | 0.119 | 0.124 | 0.104 | -0.134 |
|  | (0.000) | (0.147) | (0.723) | (0.653) | (0.642) | (0.697) | (0.613) |
| *N* | 2108 | 2100 | 2037 | 2037 | 2037 | 2037 | 2037 |
| *R*^2^ | 0.021 | 0.321 | 0.376 | 0.377 | 0.377 | 0.377 | 0.370 |
| adj. *R*^2^ | 0.021 | 0.318 | 0.371 | 0.371 | 0.371 | 0.371 | 0.364 |
| *AIC* | -5856.5 | -6607.7 | -6669.0 | -6668.3 | -6666.3 | -6665.4 | -6643.3 |

*Sources*: Ministry of Health, IBGE, TSE, Facebook Movement Range; authors’ calculations.

^*^ *p* < 0.10, ^**^ *p* < 0.05, ^***^ *p* < 0.01, ^****^ *p* < 0.001

*Note*: OLS model.
