# Supplementary material for: Investigating the ‘Bolsonaro effect’ on the spread of the Covid-19 pandemic: An empirical analysis of observational data in Brazil
Source: PLoS One. 2024 Apr 18;19(4):e0288894. doi: 10.1371/journal.pone.0288894 (PMC11025779; doi:10.1371/journal.pone.0288894)
Supplement: S5 Table — Sources: Ministry of Health, IBGE, TSE; authors’ calculations. * p < 0.10, ** p < 0.05, *** p < 0.01, **** p < 0.001. Note: Negative Binomial (NB) model, except for the column 3b and 6b (Poisson State fixed effect). (DOCX) [file pone.0288894.s005.docx]

**S5 Table. Factors associated with vaccination rates – detailed results**

**(cumulative data: from January 2021 to December 2022)**

|  |  | | | First dose | | | Full schedule | | | | | |
| --- | --- | --- | --- | --- | --- | --- | --- | --- | --- | --- | --- | --- |
|  | | (1) | (2) | | (3a) | (3b)  Poisson fe | | (4) | (5) | (6a) | (6b)  Poisson fe |  |
|  | |  |  | |  |  | |  |  |  |  |  |
| **Vote for Bolsonaro**  **(1^st^ round 2018)** | | **0.126^****^** | **-0.143^****^** | | **-0.155^****^** | **-0.230^****^** | | **0.205^****^** | **-0.231^****^** | **-0.238^****^** | **-0.315^****^** |  |
|  | | (0.000) | (0.000) | | (0.000) | (0.001) | | (0.000) | (0.000) | (0.000) | (0.000) |  |
|  | |  |  | |  |  | |  |  |  |  |  |
| Poverty level (Auxilio E.) | |  | 1.753^****^ | | 1.880^****^ | 1.926^****^ | |  | 1.649^****^ | 1.821^****^ | 1.843^****^ |  |
|  | |  | (0.000) | | (0.000) | (0.000) | |  | (0.000) | (0.000) | (0.000) |  |
| Age (log) | |  | 0.874^****^ | | 0.723^****^ | 0.665^****^ | |  | 1.186^****^ | 0.875^****^ | 0.815^****^ |  |
|  | |  | (0.000) | | (0.000) | (0.000) | |  | (0.000) | (0.000) | (0.000) |  |
| Race (White) | |  | 0.213^****^ | | 0.233^****^ | 0.203^****^ | |  | 0.264^****^ | 0.286^****^ | 0.236^****^ |  |
|  | |  | (0.000) | | (0.000) | (0.000) | |  | (0.000) | (0.000) | (0.000) |  |
| Sex (Male) | |  | -0.675^****^ | | -0.571^***^ | -1.079^***^ | |  | -0.700^****^ | -0.431^**^ | -0.989^**^ |  |
|  | |  | (0.000) | | (0.005) | (0.007) | |  | (0.000) | (0.049) | (0.018) |  |
| Education (Higher) | |  | 0.227^*^ | | 0.522^****^ | -0.163 | |  | 0.198 | 0.665^****^ | -0.188 |  |
|  | |  | (0.070) | | (0.001) | (0.525) | |  | (0.148) | (0.000) | (0.539) |  |
| GDP/cap (log) | |  | 0.0875^****^ | | 0.0834^****^ | 0.0867^****^ | |  | 0.0947^****^ | 0.0908^****^ | 0.0927^****^ |  |
|  | |  | (0.000) | | (0.000) | (0.000) | |  | (0.000) | (0.000) | (0.000) |  |
| Life Expectancy (log) | |  | 0.635^****^ | | 0.627^****^ | 0.277^***^ | |  | 0.683^****^ | 0.662^****^ | 0.276^**^ |  |
|  | |  | (0.000) | | (0.000) | (0.008) | |  | (0.000) | (0.000) | (0.024) |  |
|  | |  |  | |  |  | |  |  |  |  |  |
| Nb. Doctors (/100K h) | |  |  | | 0.0294 | 0.0742^**^ | |  |  | 0.0118 | 0.0652^*^ |  |
|  | |  |  | | (0.291) | (0.028) | |  |  | (0.693) | (0.062) |  |
| Density (log) | |  |  | | -0.0058^***^ | -0.00353 | |  |  | -0.0063^***^ | -0.00279 |  |
|  | |  |  | | (0.006) | (0.489) | |  |  | (0.006) | (0.626) |  |
| Area (Rural) | |  |  | | 0.0234^*^ | 0.0299 | |  |  | 0.0443^***^ | 0.0539^**^ |  |
|  | |  |  | | (0.098) | (0.257) | |  |  | (0.004) | (0.043) |  |
| Migration (Migrant) | |  |  | | -0.0146 | -0.00955 | |  |  | -0.0495^***^ | -0.0331 |  |
|  | |  |  | | (0.382) | (0.848) | |  |  | (0.006) | (0.538) |  |
| Job (Commuting) | |  |  | | 0.211^****^ | 0.142^****^ | |  |  | 0.285^****^ | 0.173^****^ |  |
|  | |  |  | | (0.000) | (0.000) | |  |  | (0.000) | (0.000) |  |
| Dwelling (Overcrowding) | |  |  | | -0.085^****^ | -0.127 | |  |  | -0.183^****^ | -0.205^**^ |  |
|  | |  |  | | (0.000) | (0.118) | |  |  | (0.000) | (0.028) |  |
| Location (Favela) | |  |  | | -0.252^****^ | -0.174^****^ | |  |  | -0.282^****^ | -0.183^***^ |  |
|  | |  |  | | (0.000) | (0.001) | |  |  | (0.000) | (0.002) |  |
| Job (Informal) | |  |  | | -0.186^****^ | -0.156 | |  |  | -0.222^****^ | -0.179 |  |
|  | |  |  | | (0.000) | (0.108) | |  |  | (0.000) | (0.109) |  |
|  | |  |  | |  |  | |  |  |  |  |  |
| Constant | | 4.462^****^ | -2.196^****^ | | -1.680^****^ |  | | 4.334^****^ | -3.608^****^ | -2.613^****^ |  |  |
|  | | (0.000) | (0.000) | | (0.000) |  | | (0.000) | (0.000) | (0.000) |  |  |
|  | |  |  | |  |  | |  |  |  |  |  |
| Lnalpha | | -3.773^****^ | -4.793^****^ | | -4.890^****^ |  | | -3.418^****^ | -4.522^****^ | -4.657^****^ |  |  |
|  | | (0.000) | (0.000) | | (0.000) |  | | (0.000) | (0.000) | (0.000) |  |  |
| *N* | | 5568 | 5340 | | 5269 | 5268 | | 5568 | 5340 | 5269 | 5268 |  |
| pseudo *R*^2^ | | 0.002 | 0.069 | | 0.074 |  | | 0.004 | 0.081 | 0.088 |  |  |
| *AIC* | | 47149.2 | 42171.1 | | 41409.1 | 41069.3 | | 47619.2 | 42113.6 | 41252.6 | 40890.5 |  |

*Sources*: Ministry of Health, IBGE, TSE; authors’ calculations.

^*^ *p* < 0.10, ^**^ *p* < 0.05, ^***^ *p* < 0.01, ^****^ *p* < 0.001

*Note*: Negative Binomial (NB) model, except for the column 3b and 6b (Poisson State fixed effect).
