# Supplementary material for: Investigating the ‘Bolsonaro effect’ on the spread of the Covid-19 pandemic: An empirical analysis of observational data in Brazil
Source: PLoS One. 2024 Apr 18;19(4):e0288894. doi: 10.1371/journal.pone.0288894 (PMC11025779; doi:10.1371/journal.pone.0288894)
Supplement: S6 Table — Sources: Ministry of Health, IBGE, TSE; authors’ calculations. * p < 0.10, ** p < 0.05, *** p < 0.01, **** p < 0.001. Note: Negative Binomial (NB) model. (DOCX) [file pone.0288894.s006.docx]

**S6 Table**. **Factors associated with Covid-19 and other disease vaccination rate (2017-2022)**

|  | **(1)** | **(2)** | |  | | **(3)** | | **(4)** | **(5)** | **(6)** | **(7)** | |
| --- | --- | --- | --- | --- | --- | --- | --- | --- | --- | --- | --- | --- |
|  | **Covid-19** | | **All other Vaccines** | | | | | | | | | |
|  | **1^st^ dose** | **2^nd^ dose** | |  | **2017-2018** | | **2019** | | **2020** | **2021** | | **2022** |
|  |  |  | |  |  | |  | |  |  | |  |
| **Bolsonaro (1^st^ round 2018)** | **-0.155^****^** | **-0.238^****^** | |  | **-0.0133** | | **-0.0429** | | **-0.103^**^** | **-0.158^****^** | | **-0.148^****^** |
|  | **(0.000)** | **(0.000)** | |  | **(0.668)** | | **(0.223)** | | **(0.020)** | **(0.001)** | | **(0.000)** |
|  |  |  | |  |  | |  | |  |  | |  |
| Poverty (Auxilio) | 1.880^****^ | 1.821^****^ | |  | 0.113 | | 0.156^*^ | | 0.0256 | 0.240^**^ | | 0.183^**^ |
|  | (0.000) | (0.000) | |  | (0.127) | | (0.060) | | (0.808) | (0.030) | | (0.031) |
| Age (log) | 0.723^****^ | 0.875^****^ | |  | 0.193^***^ | | 0.0926 | | 0.0504 | 0.0863 | | 0.136^*^ |
|  | (0.000) | (0.000) | |  | (0.007) | | (0.257) | | (0.624) | (0.429) | | (0.100) |
| Race (White) | 0.233^****^ | 0.286^****^ | |  | 0.0988^****^ | | 0.198^****^ | | 0.222^****^ | 0.245^****^ | | 0.0940^****^ |
|  | (0.000) | (0.000) | |  | (0.000) | | (0.000) | | (0.000) | (0.000) | | (0.000) |
| Sex (Male) | -0.571^***^ | -0.431^**^ | |  | 0.950^***^ | | 1.060^***^ | | 2.038^****^ | 2.010^****^ | | 1.981^****^ |
|  | (0.005) | (0.049) | |  | (0.002) | | (0.002) | | (0.000) | (0.000) | | (0.000) |
| Education (Higher) | 0.522^****^ | 0.665^****^ | |  | -0.500^**^ | | -0.476^*^ | | -0.569^*^ | -0.0700 | | -0.820^***^ |
|  | (0.001) | (0.000) | |  | (0.030) | | (0.068) | | (0.083) | (0.841) | | (0.002) |
| GDP/cap (log) | 0.0834^****^ | 0.0908^****^ | |  | 0.0173^**^ | | -0.00998 | | -0.00732 | -0.00205 | | -0.00774 |
|  | (0.000) | (0.000) | |  | (0.015) | | (0.212) | | (0.467) | (0.848) | | (0.340) |
| Life Expectancy (log) | 0.627^****^ | 0.662^****^ | |  | 0.350^**^ | | 0.0330 | | 0.359^*^ | 0.0332 | | -0.183 |
|  | (0.000) | (0.000) | |  | (0.012) | | (0.835) | | (0.073) | (0.875) | | (0.251) |
|  |  |  | |  |  | |  | |  |  | |  |
| Nb. Doctors (/100K h) | 0.0294 | 0.0118 | |  | -0.0000517 | | 0.0000363 | | 0.0000681 | -0.0000959 | | -0.000133^***^ |
|  | (0.291) | (0.693) | |  | (0.218) | | (0.442) | | (0.245) | (0.143) | | (0.007) |
| Density (log) | -0.00579^***^ | -0.00627^***^ | |  | -0.00957^***^ | | -0.0267^****^ | | -0.0261^****^ | -0.0237^****^ | | -0.0127^****^ |
|  | (0.006) | (0.006) | |  | (0.002) | | (0.000) | | (0.000) | (0.000) | | (0.000) |
| Area (Rural) | 0.0234^*^ | 0.0443^***^ | |  | 0.0718^****^ | | 0.0479^**^ | | 0.0844^***^ | 0.00245 | | 0.0986^****^ |
|  | (0.098) | (0.004) | |  | (0.001) | | (0.046) | | (0.005) | (0.939) | | (0.000) |
| Migration (Migrant) | -0.0146 | -0.0495^***^ | |  | 0.0822^****^ | | 0.138^****^ | | 0.0913^**^ | 0.0653^*^ | | 0.119^****^ |
|  | (0.382) | (0.006) | |  | (0.001) | | (0.000) | | (0.010) | (0.082) | | (0.000) |
| Job (Commuting) | 0.211^****^ | 0.285^****^ | |  | -0.0495 | | 0.00394 | | 0.0767 | 0.140^***^ | | 0.0245 |
|  | (0.000) | (0.000) | |  | (0.168) | | (0.923) | | (0.137) | (0.010) | | (0.550) |
| Dwelling (Overcrowding) | -0.0847^****^ | -0.183^****^ | |  | -0.216^****^ | | -0.279^****^ | | -0.547^****^ | -0.504^****^ | | -0.474^****^ |
|  | (0.000) | (0.000) | |  | (0.000) | | (0.000) | | (0.000) | (0.000) | | (0.000) |
| Location (Favela) | -0.252^****^ | -0.282^****^ | |  | -0.191^***^ | | -0.131^*^ | | -0.254^**^ | -0.212^**^ | | -0.436^****^ |
|  | (0.000) | (0.000) | |  | (0.007) | | (0.097) | | (0.011) | (0.045) | | (0.000) |
| Job (Informal) | -0.186^****^ | -0.222^****^ | |  | -0.110^**^ | | -0.0727 | | 0.0404 | -0.00740 | | -0.120^*^ |
|  | (0.000) | (0.000) | |  | (0.046) | | (0.245) | | (0.609) | (0.929) | | (0.056) |
|  |  |  | |  |  | |  | |  |  | |  |
| Constant | -1.680^****^ | -2.613^****^ | |  | 1.465^**^ | | 3.334^****^ | | 1.387 | 2.548^***^ | | 3.526^****^ |
|  | (0.000) | (0.000) | |  | (0.018) | | (0.000) | | (0.118) | (0.007) | | (0.000) |
|  |  |  | |  |  | |  | |  |  | |  |
| Lnalpha | -4.890^****^ | -4.657^****^ | |  | -3.521^****^ | | -3.189^****^ | | -2.627^****^ | -2.512^****^ | | -3.198^****^ |
|  | (0.000) | (0.000) | |  | (0.000) | | (0.000) | | (0.000) | (0.000) | | (0.000) |
| *N* | 5269 | 5269 | |  | 5269 | | 5269 | | 5269 | 5269 | | 5269 |
| *R*^2^ |  |  | |  |  | |  | |  |  | |  |
| adj. *R*^2^ |  |  | |  |  | |  | |  |  | |  |
| pseudo *R*^2^ | 0.074 | 0.088 | |  | 0.019 | | 0.017 | | 0.023 | 0.017 | | 0.020 |
| *AIC* | 41409.1 | 41252.6 | |  | 44552.6 | | 45857.4 | | 47538.7 | 47181.3 | | 45028.5 |

*Sources*: Ministry of Health, IBGE, TSE; authors’ calculations.

^*^ *p* < 0.10, ^**^ *p* < 0.05, ^***^ *p* < 0.01, ^****^ *p* < 0.001

*Note*: Negative Binomial (NB) model.
