# Supplementary material for: Investigating the ‘Bolsonaro effect’ on the spread of the Covid-19 pandemic: An empirical analysis of observational data in Brazil
Source: PLoS One. 2024 Apr 18;19(4):e0288894. doi: 10.1371/journal.pone.0288894 (PMC11025779; doi:10.1371/journal.pone.0288894)
Supplement: S7 Table — Sources: Ministry of Health, IBGE, TSE; authors’ calculations. Note: p-values in parentheses p < 0.10, ** p < 0.05, *** p < 0.01, **** p < 0.001. Negative Binomial model. The reference to compute excess mortality for each municipio is the average for the three-year period 2017–2019. For the first column, the Covid-19 mortality rate is the official figure (Ministry of Health). (DOCX) [file pone.0288894.s007.docx]

**S7 Table. Factors associated with excess mortality (cumulative data: Jan 2020 – Aug 2022)**

|  | (1) | (2) | (3) | (4) | (1) | (2) | (3) | (4) |  |  |
| --- | --- | --- | --- | --- | --- | --- | --- | --- | --- | --- |
|  | Jan-  Dec2020 | Jan20-  Dec21 | Jan20-  Apr22 | Jan20-  Aug22 | Jan-  Dec2020 | Jan20-  Dec21 | Jan20-  Apr22 | Jan20-  Aug22 | *Jan20-*  *Apr22* | *Jan20-*  *Jul22* |
|  | **Excess mortality on total death** | | | | **Excess mortality on natural death** | | | | ***Official Covid data*** | |
|  |  |  |  |  |  |  |  |  |  |  |
| **Bolsonaro** | **0.700^***^** | **0.791^****^** | **0.716^****^** | **0.591^****^** | **0.757^***^** | **0.868^****^** | **0.784^****^** | **0.663^****^** | ***0.572^****^*** | ***0.844^****^*** |
| (1^st^ round 2018) | (0.004) | (0.000) | (0.000) | (0.000) | (0.001) | (0.000) | (0.000) | (0.000) | *(0.000)* | *(0.000)* |
|  |  |  |  |  |  |  |  |  |  |  |
| Poverty (Auxilio) | 3.490^****^ | 3.145^****^ | 3.112^****^ | 3.168^****^ | 3.379^****^ | 3.011^****^ | 2.995^****^ | 3.007^****^ | *1.542^****^* | *2.493^****^* |
|  | (0.000) | (0.000) | (0.000) | (0.000) | (0.000) | (0.000) | (0.000) | (0.000) | *(0.000)* | *(0.000)* |
| Age (log) | 2.223^****^ | 1.381^****^ | 1.440^****^ | 1.390^****^ | 2.374^****^ | 1.416^****^ | 1.444^****^ | 1.379^****^ | *2.757^****^* | *2.366^****^* |
|  | (0.000) | (0.000) | (0.000) | (0.000) | (0.000) | (0.000) | (0.000) | (0.000) | *(0.000)* | *(0.000)* |
| Race (White) | -0.440^**^ | 0.0509 | 0.115 | 0.339^***^ | -0.516^***^ | -0.00576 | 0.0576 | 0.255^**^ | *-0.148^***^* | *0.125^***^* |
|  | (0.024) | (0.662) | (0.282) | (0.004) | (0.006) | (0.959) | (0.573) | (0.024) | *(0.009)* | *(0.010)* |
| Sex (Male) | 0.373 | 0.188 | 0.0373 | -0.507 | 0.327 | -0.111 | -0.368 | -0.853 | *1.088* | *-1.873^***^* |
|  | (0.880) | (0.899) | (0.978) | (0.737) | (0.891) | (0.937) | (0.775) | (0.550) | *(0.148)* | *(0.002)* |
| Education (Higher) | -0.0628 | 1.494 | 1.312 | 1.618 | -0.658 | 1.150 | 0.914 | 1.115 | *1.298^**^* | *1.228^**^* |
|  | (0.973) | (0.190) | (0.209) | (0.163) | (0.711) | (0.294) | (0.360) | (0.311) | *(0.023)* | *(0.010)* |
| GDP/cap (log) | 0.106^*^ | 0.113^***^ | 0.0992^***^ | 0.105^***^ | 0.118^**^ | 0.122^****^ | 0.108^****^ | 0.114^****^ | *0.151^****^* | *0.145^****^* |
|  | (0.064) | (0.001) | (0.002) | (0.004) | (0.033) | (0.000) | (0.000) | (0.001) | *(0.000)* | *(0.000)* |
| Life Expectancy (log) | -1.633 | -0.0501 | 0.0714 | -0.0703 | -1.235 | -0.0152 | 0.116 | 0.0228 | *0.931^***^* | *1.372^****^* |
|  | (0.138) | (0.940) | (0.908) | (0.919) | (0.245) | (0.981) | (0.844) | (0.972) | *(0.007)* | *(0.000)* |
|  |  |  |  |  |  |  |  |  |  |  |
| Nb. Doctors (/100K h) | -0.430 | -0.177 | -0.141 | -0.170 | -0.267 | -0.188 | -0.140 | -0.169 | *0.0925* | *0.000628* |
|  | (0.286) | (0.413) | (0.489) | (0.433) | (0.496) | (0.392) | (0.487) | (0.423) | *(0.417)* | *(0.995)* |
| Density (log) | -0.0105 | -0.0212 | -0.0253^*^ | -0.0327^**^ | 0.00243 | -0.0120 | -0.0159 | -0.0226 | *-0.0377^****^* | *-0.0296^****^* |
|  | (0.667) | (0.158) | (0.067) | (0.032) | (0.918) | (0.406) | (0.226) | (0.120) | *(0.000)* | *(0.000)* |
| Area (Rural) | 0.0236 | -0.290^***^ | -0.242^**^ | -0.249^**^ | 0.0175 | -0.322^****^ | -0.275^***^ | -0.296^***^ | *-0.428^****^* | *-0.517^****^* |
|  | (0.889) | (0.004) | (0.010) | (0.017) | (0.914) | (0.001) | (0.002) | (0.003) | *(0.000)* | *(0.000)* |
| Migration (Migrant) | 0.516^**^ | 0.423^****^ | 0.351^***^ | 0.521^****^ | 0.422^**^ | 0.365^***^ | 0.295^***^ | 0.452^****^ | *0.628^****^* | *0.407^****^* |
|  | (0.011) | (0.000) | (0.002) | (0.000) | (0.032) | (0.002) | (0.006) | (0.000) | *(0.000)* | *(0.000)* |
| Job (Commuting) | 0.183 | 0.0622 | 0.0991 | 0.118 | 0.176 | 0.0611 | 0.106 | 0.125 | *-0.0574* | *0.122^*^* |
|  | (0.521) | (0.720) | (0.534) | (0.505) | (0.522) | (0.714) | (0.487) | (0.456) | *(0.509)* | *(0.093)* |
| Dwelling (o/crowd) | 0.491^*^ | 0.295^*^ | 0.249 | 0.302^*^ | 0.599^**^ | 0.337^**^ | 0.278^*^ | 0.323^**^ | *0.718^****^* | *0.623^****^* |
|  | (0.071) | (0.075) | (0.102) | (0.074) | (0.022) | (0.034) | (0.056) | (0.045) | *(0.000)* | *(0.000)* |
| Location (Favela) | 0.976^*^ | 0.193 | 0.0604 | -0.0416 | 1.028^*^ | 0.195 | 0.0583 | -0.0306 | *0.612^****^* | *0.486^****^* |
|  | (0.087) | (0.572) | (0.847) | (0.904) | (0.063) | (0.551) | (0.844) | (0.926) | *(0.000)* | *(0.001)* |
| Job (Informal) | -0.720^*^ | -0.624^**^ | -0.615^**^ | -0.665^**^ | -0.630 | -0.500^*^ | -0.501^**^ | -0.522^**^ | *-0.591^****^* | *-0.140* |
|  | (0.099) | (0.021) | (0.013) | (0.015) | (0.134) | (0.053) | (0.034) | (0.045) | *(0.000)* | *(0.217)* |
|  |  |  |  |  |  |  |  |  |  |  |
| Constant | 2.036 | -0.798 | -1.104 | -0.157 | -0.257 | -0.996 | -1.186 | -0.412 | *-10.15^****^* | *-9.680^****^* |
|  | (0.673) | (0.787) | (0.685) | (0.959) | (0.956) | (0.725) | (0.647) | (0.886) | *(0.000)* | *(0.000)* |
|  |  |  |  |  |  |  |  |  |  |  |
| Lnalpha | 0.973^****^ | -0.0215 | -0.191^****^ | 0.0137 | 0.906^****^ | -0.106^****^ | -0.288^****^ | -0.0903^****^ | *-1.419^****^* | *-1.777^****^* |
|  | (0.000) | (0.265) | (0.000) | (0.474) | (0.000) | (0.000) | (0.000) | (0.000) | *(0.000)* | *(0.000)* |
| *N* | 5269 | 5269 | 5269 | 5269 | 5269 | 5269 | 5269 | 5269 | *5269* | *5269* |
| *R*^2^ |  |  |  |  |  |  |  |  |  |  |
| adj. *R*^2^ |  |  |  |  |  |  |  |  |  |  |
| pseudo *R*^2^ | 0.002 | 0.005 | 0.005 | 0.005 | 0.002 | 0.005 | 0.005 | 0.005 | *0.031* | *0.047* |
| *AIC* | 55343.5 | 70850.2 | 72816.2 | 73155.1 | 55481.5 | 70622.7 | 72513.7 | 73014.2 | *64275.6* | *62675.3* |

*Sources*: Ministry of Health, IBGE, TSE; authors’ calculations.

*Note*: *p*-values in parentheses *p* < 0.10, ^**^ *p* < 0.05, ^***^ *p* < 0.01, ^****^ *p* < 0.001

Negative Binomial model. The reference to compute excess mortality for each *municipio* is the average for the three-year period 2017-2019.

For the first column, the Covid-19 mortality rate is the official figure (Ministry of Health).
