# Supplementary material for: Investigating the ‘Bolsonaro effect’ on the spread of the Covid-19 pandemic: An empirical analysis of observational data in Brazil
Source: PLoS One. 2024 Apr 18;19(4):e0288894. doi: 10.1371/journal.pone.0288894 (PMC11025779; doi:10.1371/journal.pone.0288894)
Supplement: S8 Table — Sources: Ministry of Health, IBGE, TSE; authors’ calculations. p-values in parentheses p < 0.10, ** p < 0.05, *** p < 0.01, **** p < 0.001. Note: Negative Binomial (NB) model. The control variables are always the same (those considered in Table 4) for each of the three specifications considered here (with only the percentage of votes for Bolsonaro in the first round; with only the percentage of votes for Bolsonaro in the second round; with the percentage of votes for Bolsonaro in the first round and the difference 2nd -1st round). (DOCX) [file pone.0288894.s008.docx]

**S8 Table**. **Further exploration of the ‘Bolsonaro effect’ on the Covid-19 mortality rate (2018 election)**

|  |  | Q1 | Q2 | Q3 | Q4 | Q5 | Q6 | Q7 | Q8 | Q9 | Q10 | Q11 |
| --- | --- | --- | --- | --- | --- | --- | --- | --- | --- | --- | --- | --- |
|  | *Cumulative*  *data at the*  *end of Oct.*  *2021* | May-July 2020 | Aug-Oct 2020 | Nov 2020-Jan 2021 | Feb-Apr 2021 | May-Jul 2021 | Aug-Oct 2021 | Nov 2021-Jan 2022 | Feb-Apr 2022 | May-Jul 2022 | Aug-Oct 2022 | Nov-Dec 2022 |
|  |  |  |  |  |  |  |  |  |  |  |  |  |
| Vote for Bolsonaro  1^st^ round | *0.836^****^* | 1.370^****^ | 1.242^****^ | 1.108^****^ | 0.604^****^ | 0.461^****^ | 1.415^****^ | 0.721*** | -0.285 | 1.189**** | 1.389**** | 0.769 |
|  |  |  |  |  |  |  |  |  |  |  |  |  |
| Pseudo R2 | *0.040* | 0.014 | 0.007 | 0.010 | 0.018 | 0.021 | 0.019 | 0.014 | 0.017 | 0.012 | 0.013 | 0.012 |
|  |  |  |  |  |  |  |  |  |  |  |  |  |
|  |  |  |  |  |  |  |  |  |  |  |  |  |
| Vote for Bolsonaro  2nd round | *0.691^****^* | 0.941^****^ | 0.893^****^ | 0.762^****^ | 0.416^****^ | 0.511^****^ | 1.618^****^ | 0.773^****^ | -0.491^**^ | 0.961^***^ | 1.644^****^ | 1.031^**^ |
|  |  |  |  |  |  |  |  |  |  |  |  |  |
| Pseudo R2 | *0.040* | 0.013 | 0.007 | 0.009 | 0.018 | 0.021 | 0.020 | 0.014 | 0.017 | 0.012 | 0.013 | 0.012 |
|  |  |  |  |  |  |  |  |  |  |  |  |  |
|  |  |  |  |  |  |  |  |  |  |  |  |  |
| Vote for Bolsonaro  1^st^ round | *0.862^****^* | 1.578^****^ | 1.425^****^ | 1.311^****^ | 0.714^****^ | 0.341^**^ | 1.023^****^ | 0.563^**^ | -0.0286 | 1.226^****^ | 0.775^*^ | 0.253 |
|  |  |  |  |  |  |  |  |  |  |  |  |  |
| Difference vote  for Bolsonaro  2^nd^ -1^st^ round | *-0.318* | -2.916^****^ | -2.300^****^ | -2.565^****^ | -1.326^***^ | 1.463^****^ | 5.095^****^ | 1.961^**^ | -2.776^****^ | -0.461 | 6.503^****^ | 5.129^****^ |
|  |  |  |  |  |  |  |  |  |  |  |  |  |
| Pseudo R2 | *0.040* | 0.014 | 0.008 | 0.010 | 0.018 | 0.021 | 0.020 | 0.014 | 0.018 | 0.012 | 0.014 | 0.012 |
|  |  |  |  |  |  |  |  |  |  |  |  |  |
| Control variables | Yes | Yes | Yes | Yes | Yes | Yes | Yes | Yes | Yes | Yes | Yes | Yes |
| N | *5269* | 5269 | 5269 | 5269 | 5269 | 5269 | 5269 | 5269 | 5269 | 5269 | 5269 | 5269 |

*Sources*: Ministry of Health, IBGE, TSE; authors’ calculations.

*p*-values in parentheses *p* < 0.10, ^**^ *p* < 0.05, ^***^ *p* < 0.01, ^****^ *p* < 0.001

*Note*: Negative Binomial (NB) model. The control variables are always the same (those considered in table 4) for each of the three specifications considered here (with only the percentage of votes for Bolsonaro in the first round; with only the percentage of votes for Bolsonaro in the second round; with the percentage of votes for Bolsonaro in the first round and the difference 2^nd^ -1^st^ round)
