# Supplementary material for: Investigating the ‘Bolsonaro effect’ on the spread of the Covid-19 pandemic: An empirical analysis of observational data in Brazil
Source: PLoS One. 2024 Apr 18;19(4):e0288894. doi: 10.1371/journal.pone.0288894 (PMC11025779; doi:10.1371/journal.pone.0288894)
Supplement: S9 Table — Sources: Ministry of Health, IBGE, TSE; authors’ calculations. p-values in parentheses p < 0.10, ** p < 0.05, *** p < 0.01, **** p < 0.001. Note: Negative Binomial (NB) model. The control variables are always the same (those considered in Table 4) for each of the two specifications considered here (with only the percentage of votes for Bolsonaro in the first round in 2018; with only the percentage of votes for Bolsonaro in the first round in 2022. (DOCX) [file pone.0288894.s009.docx]

**S9 Table**. **The ‘Bolsonaro effect’ on Covid-19 mortality, social distancing and vaccination (2022 election)** (cumulative data)

|  | (1) | (2) | (3) | (4) | (5) | (6) | (7) | (8) | (9) | (10) | (11) | (12) |
| --- | --- | --- | --- | --- | --- | --- | --- | --- | --- | --- | --- | --- |
|  | End Apr20 | Jul20 | Oct20 | Jan21 | Apr21 | Jul21 | Oct21 | Jan22 | Apr22 | Jul22 | Oct22 | Dec22 |
| **Mortality rate** |  |  |  |  |  |  |  |  |  |  |  |  |
| **Bolsonaro 2018T1** | 0.388 | 1.349^****^ | 1.349^****^ | 1.267^****^ | 0.928^****^ | 0.800^****^ | 0.836^****^ | 0.833^****^ | 0.572^****^ | 0.844^****^ | 0.856^****^ | 0.855^****^ |
|  | (0.589) | (0.000) | (0.000) | (0.000) | (0.000) | (0.000) | (0.000) | (0.000) | (0.000) | (0.000) | (0.000) | (0.000) |
| **Bolsonaro 2022T1** | 0.427 | 1.629^****^ | 1.454^****^ | 1.322^****^ | 1.053^****^ | 0.889^****^ | 0.918^****^ | 0.921^****^ | 0.579^****^ | 0.930^****^ | 0.946^****^ | 0.947^****^ |
|  | (0.568) | (0.000) | (0.000) | (0.000) | (0.000) | (0.000) | (0.000) | (0.000) | (0.000) | (0.000) | (0.000) | (0.000) |
| **Mobility rate** |  |  |  |  |  |  |  |  |  |  |  |  |
| **Bolsonaro 2018T1** | 0.0336^****^ | 0.0625^****^ | 0.0517^****^ | 0.0391^****^ | 0.0529^****^ | 0.0640^****^ | 0.0607^****^ |  |  |  |  |  |
|  | (0.000) | (0.000) | (0.000) | (0.000) | (0.000) | (0.000) | (0.000) |  |  |  |  |  |
| **Bolsonaro 2022T1** | 0.0347^****^ | 0.0584^****^ | 0.0531^****^ | 0.0403^****^ | 0.0526^****^ | 0.0620^****^ | 0.0616^****^ |  |  |  |  |  |
|  | (0.000) | (0.000) | (0.000) | (0.000) | (0.000) | (0.000) | (0.000) |  |  |  |  |  |
| **Vaccination rate** (full vaccination) |  |  |  |  |  |  |  |  |  |  |  |  |
| **Bolsonaro 2018T1** |  |  |  |  |  | -0.121^****^ | -0.177^****^ | -0.167^****^ | -0.238^****^ | -0.236^****^ | -0.237^****^ | -0.238^****^ |
|  |  |  |  |  |  | (0.000) | (0.000) | (0.000) | (0.000) | (0.000) | (0.000) | (0.000) |
| **Bolsonaro 2022T1** |  |  |  |  |  | -0.0980^***^ | -0.156^****^ | -0.175^****^ | -0.252^****^ | -0.253^****^ | -0.254^****^ | -0.255^****^ |
|  |  |  |  |  |  | (0.004) | (0.000) | (0.000) | (0.000) | (0.000) | (0.000) | (0.000) |
| **Other Controls** | Yes | Yes | Yes | Yes | Yes | Yes | Yes | Yes | Yes | Yes | Yes | Yes |

*Sources*: Ministry of Health, IBGE, TSE; authors’ calculations.

*p*-values in parentheses *p* < 0.10, ^**^ *p* < 0.05, ^***^ *p* < 0.01, ^****^ *p* < 0.001

*Note*: Negative Binomial (NB) model. The control variables are always the same (those considered in Table 4) for each of the two specifications considered here (with only the percentage of votes for Bolsonaro in the first round in 2018 ; with only the percentage of votes for Bolsonaro in the first round in 2022.
