# Supplementary material for: Investigating the ‘Bolsonaro effect’ on the spread of the Covid-19 pandemic: An empirical analysis of observational data in Brazil
Source: PLoS One. 2024 Apr 18;19(4):e0288894. doi: 10.1371/journal.pone.0288894 (PMC11025779; doi:10.1371/journal.pone.0288894)
Supplement: S10 Table — Sources: Ministry of Health, IBGE, TSE; authors’ calculations. p-values in parentheses p < 0.10, ** p < 0.05, *** p < 0.01, **** p < 0.001. Note: Negative Binomial (NB) model. The control variables are always the same (those considered in Table 4; see also S9 Table) for each of the specifications considered. (DOCX) [file pone.0288894.s010.docx]

**S10 Table**. **The ‘Bolsonaro effect’ on Covid-19 mortality, mobility and vaccination rates controlling for preferences (cumulative data)**

|  | (1) | (2) | (3) | (4) | (5) | (6) | (7) | (8) | (9) | (10) | (11) | (12) |
| --- | --- | --- | --- | --- | --- | --- | --- | --- | --- | --- | --- | --- |
|  | End Apr20 | Jul20 | Oct20 | Jan21 | Apr21 | Jul21 | Oct21 | Jan22 | Apr22 | Jul22 | Oct22 | Dec22 |
| **Mortality rate** |  |  |  |  |  |  |  |  |  |  |  |  |
| **Bolsonaro 2018T1** | 0.388 | 1.349^****^ | 1.349^****^ | 1.267^****^ | 0.928^****^ | 0.800^****^ | 0.836^****^ | 0.833^****^ | 0.572^****^ | 0.844^****^ | 0.856^****^ | 0.855^****^ |
| (reference) | (0.589) | (0.000) | (0.000) | (0.000) | (0.000) | (0.000) | (0.000) | (0.000) | (0.000) | (0.000) | (0.000) | (0.000) |
|  |  |  |  |  |  |  |  |  |  |  |  |  |
| **Bolsonaro 2018T1** | 2.115^**^ | 2.278^****^ | 2.001^****^ | 1.865^****^ | 1.409^****^ | 0.932^****^ | 0.876^****^ | 0.866^****^ | 0.556^****^ | 0.823^****^ | 0.812^****^ | 0.803^****^ |
|  | (0.022) | (0.000) | (0.000) | (0.000) | (0.000) | (0.000) | (0.000) | (0.000) | (0.000) | (0.000) | (0.000) | (0.000) |
| **Neves 2014T1** | -2.918^***^ | -1.407^****^ | -0.985^****^ | -0.916^****^ | -0.736^****^ | -0.202^**^ | -0.0602 | -0.0511 | 0.0250 | 0.0319 | 0.0660 | 0.0785 |
|  | (0.002) | (0.000) | (0.000) | (0.000) | (0.000) | (0.031) | (0.489) | (0.545) | (0.794) | (0.690) | (0.408) | (0.323) |
| **Bolsonaro 2018T1** | 0.391 | 1.366^****^ | 1.352^****^ | 1.269^****^ | 0.932^****^ | 0.803^****^ | 0.839^****^ | 0.837^****^ | 0.576^****^ | 0.848^****^ | 0.859^****^ | 0.858^****^ |
|  | (0.586) | (0.000) | (0.000) | (0.000) | (0.000) | (0.000) | (0.000) | (0.000) | (0.000) | (0.000) | (0.000) | (0.000) |
| **Vaccine 2017-2018** | 0.0331 | 0.357^***^ | 0.148^*^ | 0.143^**^ | 0.138^***^ | 0.129^***^ | 0.152^****^ | 0.166^****^ | 0.182^****^ | 0.145^****^ | 0.140^****^ | 0.136^****^ |
|  | (0.935) | (0.006) | (0.095) | (0.030) | (0.005) | (0.001) | (0.000) | (0.000) | (0.000) | (0.000) | (0.000) | (0.000) |
| **Mobility rate** |  |  |  |  |  |  |  |  |  |  |  |  |
| **Bolsonaro 2018T1** | 0.0336^****^ | 0.0625^****^ | 0.0517^****^ | 0.0391^****^ | 0.0529^****^ | 0.0640^****^ | 0.0607^****^ |  |  |  |  |  |
| (reference) | (0.000) | (0.000) | (0.000) | (0.000) | (0.000) | (0.000) | (0.000) |  |  |  |  |  |
|  |  |  |  |  |  |  |  |  |  |  |  |  |
| **Bolsonaro 2018T1** | -0.0210^*^ | 0.0165 | 0.0175 | 0.0160 | 0.0290^**^ | 0.0497^****^ | 0.0469^***^ |  |  |  |  |  |
|  | (0.076) | (0.216) | (0.202) | (0.237) | (0.029) | (0.000) | (0.001) |  |  |  |  |  |
| **Neves 2014T1** | 0.0873^****^ | 0.0733^****^ | 0.0542^****^ | 0.0365^***^ | 0.0384^***^ | 0.0230^*^ | 0.0228 |  |  |  |  |  |
|  | (0.000) | (0.000) | (0.000) | (0.006) | (0.003) | (0.093) | (0.113) |  |  |  |  |  |
| **Bolsonaro 2018T1** | 0.0336^****^ | 0.0629^****^ | 0.0520^****^ | 0.0397^****^ | 0.0531^****^ | 0.0646^****^ | 0.0611^****^ |  |  |  |  |  |
|  | (0.000) | (0.000) | (0.000) | (0.000) | (0.000) | (0.000) | (0.000) |  |  |  |  |  |
| **Vaccine 2017-2018** | 0.00848 | 0.0169^***^ | 0.0278^****^ | 0.0297^****^ | 0.0211^***^ | 0.0184^***^ | 0.0123^*^ |  |  |  |  |  |
|  | (0.144) | (0.010) | (0.000) | (0.000) | (0.002) | (0.010) | (0.096) |  |  |  |  |  |
| **Vaccination rate**  (full vaccination) |  |  |  |  |  |  |  |  |  |  |  |  |
| **Bolsonaro 2018T1** |  |  |  |  |  | -0.121^****^ | -0.177^****^ | -0.167^****^ | -0.238^****^ | -0.236^****^ | -0.237^****^ | -0.238^****^ |
| (reference) |  |  |  |  |  | (0.000) | (0.000) | (0.000) | (0.000) | (0.000) | (0.000) | (0.000) |
|  |  |  |  |  |  |  |  |  |  |  |  |  |
| **Bolsonaro 2018T1** |  |  |  |  |  | -0.224^****^ | -0.447^****^ | -0.429^****^ | -0.519^****^ | -0.513^****^ | -0.511^****^ | -0.513^****^ |
|  |  |  |  |  |  | (0.000) | (0.000) | (0.000) | (0.000) | (0.000) | (0.000) | (0.000) |
| **Neves 2014T1** |  |  |  |  |  | 0.159^****^ | 0.413^****^ | 0.400^****^ | 0.428^****^ | 0.422^****^ | 0.419^****^ | 0.420^****^ |
|  |  |  |  |  |  | (0.000) | (0.000) | (0.000) | (0.000) | (0.000) | (0.000) | (0.000) |
| **Bolsonaro 2018T1** |  |  |  |  |  | -0.121^****^ | -0.176^****^ | -0.166^****^ | -0.237^****^ | -0.235^****^ | -0.236^****^ | -0.237^****^ |
|  |  |  |  |  |  | (0.001) | (0.000) | (0.000) | (0.000) | (0.000) | (0.000) | (0.000) |
| **Vaccine 2017-2018** |  |  |  |  |  | 0.106^****^ | 0.133^****^ | 0.130^****^ | 0.139^****^ | 0.140^****^ | 0.143^****^ | 0.144^****^ |
|  |  |  |  |  |  | (0.000) | (0.000) | (0.000) | (0.000) | (0.000) | (0.000) | (0.000) |
| **Other Controls** | Yes | Yes | Yes | Yes | Yes | Yes | Yes | Yes | Yes | Yes | Yes | Yes |

*Sources*: Ministry of Health, IBGE, TSE; authors’ calculations.

*p*-values in parentheses *p* < 0.10, ^**^ *p* < 0.05, ^***^ *p* < 0.01, ^****^ *p* < 0.001

*Note*: Negative Binomial (NB) model. The control variables are always the same (those considered in Table 4; see also S9 Table) for each of the specifications considered.
