# Supplementary material for: Investigating the ‘Bolsonaro effect’ on the spread of the Covid-19 pandemic: An empirical analysis of observational data in Brazil
Source: PLoS One. 2024 Apr 18;19(4):e0288894. doi: 10.1371/journal.pone.0288894 (PMC11025779; doi:10.1371/journal.pone.0288894)
Supplement: Attachment — Submitted filename: Response to Reviewers - Investigating Bolsonaro effect.docx [file pone.0288894.s011.docx]

**Investigating the ‘*Bolsonaro effect*’ on the spread of the Covid-19 pandemic:**

**an empirical analysis of observational data in Brazil**

**Detailed answers to Reviewers**

First of all, we would like to thank the editor and the two reviewers for their positive assessment of our paper and their insightful comments. Your remarks and suggestions helped us to improve the quality of the paper. We have taken all of them into account in our revision, in general by accepting them and elaborating on the suggestions. In some rare cases, we did not develop further some suggestions, basically for space constraints. We believe that our revised manuscript has been substantially improved now, and we hope it meets the journal’s requirements for publication. In any case, we remain at your disposal for further enquiries.

Please find below our responses regarding the points you raised (in black). To facilitate the identification of our modifications, they appear in the revised draft as ‘track changes’.

Editor Comments:

The manuscript presents updated results confirming that pro-Bolsonaro municipalities had higher fatality rates than other Brazilian municipalities. The article's theme is relevant, but there are already published articles showing more cases of COVID-19 and higher mortality in Brazilian municipalities where JB had more votes. Thus, for the present article to be considered for publication, the authors need to describe this previous research and be convincing about what the present article can add to the findings of these authors. Without this effort, the present article will be seen as "more of the same." The manuscript should also debate the centrality of STF decisions in combating the pandemic and Bolsonaro's attempts to prevent social distancing measures or mandatory vaccination. The author should also explain their methodological choices and the methods proposed to make causal inferences and be more comprehensive in presenting results. Finally, the conclusion should also be refined to the broad literature about populism and democracy.

*[Our answers (in black in this document):]*

Many thanks for these comments and sound suggestions.

We made a careful review of the additional/recent literature, specifically the papers which aim at estimating the ‘Bolsonaro effect’. The revised version of the document presents more clearly the existing studies and their results, on the basis of which our paper seeks to go further and explore new avenues following different directions:

a) the time span : our estimations cover the full 3 years Covid-19 period (2020-2022), allowing to investigate the evolution of the ‘Bolsonaro effects’ over time and showing their persistence;

b) we consider not only death/cases and social distancing but also vaccination – by population categories. To our knowledge, this type of estimation using vaccination has never been explored before for the case of Brazil. Estimating the ‘Bolsonaro effect’ on social distancing and vaccination allows to investigate not only non-pharmaceutical interventions (NPI) channels (as other papers) but also the main pharmaceutical intervention (PI) channel;

c) We investigate spill over effects (on other vaccines than Covid-19) and in fact, over and above the negative impact on Covid-19 vaccination, our estimates support the hypothesis that Bolsonaro denialism impacted on compliance with the general vaccination schedule.

In addition, references and discussion have been included on the importance of the role of the STF in the manuscript. The methodological choices have been clarified. Finally, Concerning the literature about populism and democracy, the contribution of our study to this broad strand of research on populism has been outlined (see below for a more detailed answer to the reviewers).

**Reviewer #1:**

The paper is well written and has a clear and relevant research problem. The data used demonstrates that the main argument is well supported. I am not able to assess the quality of the statistical tests presented, but would like to draw attention to issues that could be improved in the text.

First, it is important to emphasize the relevance of the paper not only for what it adds when compared to the existing literature. It failed to present the reader the relevance of the work to a broader discussion, such as the impact of populist and anti-science leaders for the development of evidence-based public policies and, specifically, for facing health crises, such as Covid-19.

Regarding the works already published on the subject, I missed one that had repercussions in Brazil, that of Fernandes and Fernandes (2022), "Populism and health. An evaluation of the effects of right-wing populism on the COVID-19 pandemic in Brazil". There is a vast literature on the effects of populist leaders on democracy and its policies, and this debate should be mentioned in the paper's justification.

First, we thank the reviewer for his suggestion on the need to stress to what extent the paper is relevant. In fact, we have initially centered our analysis on the impacts on health outcomes in the context of COVID-19. But we expanded comments and references to mention the suggested literature that is somehow at the origin of the studies about political orientation and public policies (including health policy). This is of particular interest for Brazil as the consequences of political choices hit one of the most successful vaccination programs in the world.

We would like also to thank the reviewer for drawing our attention on new references, in particular Fernandes and Fernandes (2022), also suggested by Reviewer#2. It should be noted that this paper was not available when we wrote ours, but we now quote this study in the revised version of the manuscript.

Fernandes and Fernandes (2022) adds interesting elements to tackle further the spurious correlation issue. Their methodological approach adds to the previous literature in two respects. The authors conduct placebo tests for other respiratory diseases (before the pandemic outbreak).They also control for the effect of traditional right-wing votes (using the first round of the 2018 elections), to better estimate the ‘true Bolsonaro effect’. Our submitted manuscript already implemented these two strategies, and goes beyond, precisely following their recommendations for future research (for instance: p.5, ‘*Our data collection starts on February 19th and goes until December 31st, 2020…Covid response involves more strategies than NPIs. Future studies should analyze the implications.*’, p.10, ‘*Lastly, an important research topic on the effects of populism on the pandemic is related to the politics of vaccination, whose anecdotal evidence points to being another broad field of action for populist leaders*’; see also above the Response to the Editor).

More broadly, we have added clarifications on how our paper relates to the literature on the effect of populist leaders (right and left wing) on public policies. However, populism is a much debated concept in the literature (see for instance the discussion in Razafindrakoto et al., 2023) but this is not the subject of our paper. The literature on the effect of populist leaders on policies is very vast, therefore, given the space constraints, we have selected the most relevant studies that are most closely related to the management of the Covid-19 pandemic (Lasco, 2020; Ortega and Orsini, 2020; Knaul *et al.*, 2021; Bennouna *et al.*, 2021; Fernandes and Fernandes, 2022; Bertholini, 2023; Burni, Stockemer and Hackenesch, 2023).

Apart from the new papers suggested by the reviewer, we identify also another paper related to our topic and recently published in *PLOS ONE* journal (de Almeida *et al.*, 2022). Based on simple correlations at the States level, they estimate by deduction the excess fatalities due to Bolsonaro’s support at 57% (+/- 11%) at the end of 2021. Although the methodology which attributes the excess mortality to the ‘Bolsonaro effect’ could be questionable, we now quote this paper in our revised draft.

Second, the paper has not mentioned a set of existing works in the national literature that mention the centrality of STF decisions in combating the pandemic and Bolsonaro's attempts to prevent social distancing measures or mandatory vaccination. Studies such as Fernandes and Ouverney (2022) and Oliveira and Madeira (2021), among others, have presented this debate.

We took the suggestions into account. We read the papers carefully and we agree on the centrality of STF decisions. Oliveira and Madeira (2021), Fernandes and Ouverney (2022), and Ouverney and Fernandes (2022) analyze the role of various counterpowers (STF, supreme court for the former two; National Congress for the latter) to countervail Bolsonaro policies in 2020. For instance, O&M (2021) analyses the political judicialization by STF (embedded in the long run since the 1990s, called ‘the first wave of judicialization’) during Covid-19 crisis, to combat Bolsonaro’s policies through constitutional controls. By limiting Bolsonaro policies, these actions by STF and other bodies probably reduced the ‘*Bolsonaro effect*’ (without their interventions) as the more standard restrictive (health) and protective (labour market) policies were adopted at the infra-national level (by governors and mayors), mainly from the political opposition. The Covid-19 crisis reinforced the power of judiciary and legislative bodies (‘Brazilian federalism’), against the judiciary and the executive, with the president at the forefront. However, and on the contrary, the lack of coherent policies and coordination among the different government entities has also potentially the adverse consequence of increasing the ‘Bolsonaro effect’ in municipalities. At the end of day, it is not clear which of these opposed effects prevailed.

We have therefore included the references, although it should be stressed that this question is important for contextual information but the main object of our study is to identify a ‘Bolsonaro effect’ and we had to consider the space constraints. We are not seeking to disentangle the various mechanisms or procedures that may have amplified or reduced the effect of Bolsonaro denialist position (intervention by the STF or problems of institutional coordination).

In the revised version of the manuscript, we have made explicit the role played by the STF alongside subnational governments as the main obstacles to Bolsonaro's anti-science agenda, with the multiplicity of judicial debates. More globally, we have taken the opportunity of this revision to upgrade the literature review by quoting more recent papers.

Finally, the conclusions were very much centered on the empirical findings, but failed to rescue the broader debate in which it is embedded, including the one mentioned above - the impact of populist leaders on the conduct of evidence-based public policy.

We agree with the suggestions and made the changes accordingly. We have completed the conclusion in two directions: by presenting the caveats and limitations of our approach in terms of causal impact using observational data (suggested by reviewer #2); and by elaborating a little on how our paper contributes to the wider debate on populism and democracy, as suggested.

**Reviewer #2:**

The manuscript titled "Investigating the 'Bolsonaro effect' on the spread of the COVID-19 pandemic: an empirical analysis of observational data in Brazil" presents updated results confirming that pro-Bolsonaro municipalities had higher fatality rates compared to other Brazilian municipalities.
In terms of the literature review, I recommend more extensive engagement with the article "Populism and health: An evaluation of the effects of right-wing populism on the COVID-19 pandemic in Brazil," published in PLOS ONE.

We thank the reviewer for the recommendation, which was also a suggestion of reviewer#1. We have included this reference in our revised manuscript and other additional ones on the same issue. Furthermore, we have added precisions on how our paper relates to this literature (see also above the answer to reviewer#1).

To address potential bias associated with unobserved factors related to time and geography, it is advisable to include day and fixed-effects in your analysis. I discourage the use of the Negative Binomial (NB) model with fixed effects (FEs). Instead, it would be preferable to use either Linear Probability Models (LPM) or Poisson regression with fixed effects. A valuable reference for this approach is Wooldridge, J. M.'s 1999 paper, "Distribution-Free Estimation of Some Nonlinear Panel Data Models" in the Journal of Econometrics (Vol. 90, pp. 77–97).

We took into consideration this suggestion. Indeed, Negative Binomial (NB) model with fixed effects (FEs) is generally not recommended (it is a debated issue among the specialists). As a matter of fact, our paper does not estimate NB-FEs, but NBs on repeated cross-sectional successive periods (cumulative and by time-spans). As quoted in the literature (see for instance Brown and Ravallion, 2020 and 2022 in a similar setting on Covid-19 pandemic), NB is recommended for count data with overdispersion (NBs are also used by Fernandes and Fernandes, 2022).

We should also point out that we have also tested other types of model specification. For example, we considered, in a previous paper, two other options : considering the number of days since the first case (“exposure time to the pandemic”) as a control variable or considering a time trend cross-effect ($T_{it}*X_{i}$) in a panel model (Razafindrakoto et al., 2021). But this type of specification which supposes that the coefficients change linearly over time (a strong hypothesis) is not satisfactory. However, the results are always consistent with the conclusion on the persistence of a ‘Bolsonaro effect’.

The choice of the best econometric model is often debatable. Therefore, we took into consideration the suggestion and we estimate Poisson regression models with fixed effects (at State level), in order to account for unobservables invariant over time and state heterogeneity. The results are consistent with those of the NB model, confirming the ‘Bolsonaro effect’. We add these Poisson estimators in the revised tables (with the idea of carrying out a robustness check).

We provide also here for the figure 2 the corresponding figure derived from Poisson regression model. Again, we can notice the confirmation of the persistence of the ‘Bolsonaro effect’.

**Figure 2: The ‘*Bolsonaro effect*’ on the mortality rate over time (2020-2022)**

Cumulative (NB model)	Cumulative (Poisson with fixed effects)

|  | CUMULE |  |  |
| --- | --- | --- | --- |
| 2020-04-01 | -1.017 | 1.792 | 0.388 |
| 2020-07-01 | 0.861 | 1.837 | 1.349 |
| 2020-10-01 | 1.025 | 1.673 | 1.349 |
| 2021-01-01 | 1.027 | 1.506 | 1.267 |
| 2021-04-01 | 0.751 | 1.105 | 0.928 |
| 2021-07-01 | 0.656 | 0.943 | 0.8 |
| 2021-10-01 | 0.702 | 0.97 | 0.836 |
| 2022-01-01 | 0.703 | 0.963 | 0.833 |
| 2022-04-01 | 0.425 | 0.72 | 0.572 |
| 2022-07-01 | 0.721 | 0.968 | 0.844 |
| 2022-10-01 | 0.733 | 0.978 | 0.856 |
| 2022-12-01 | 0.732 | 0.977 | 0.855 |

|  | CUMULE |  |  |
| --- | --- | --- | --- |
| 2020-04-01 | -0.827 | 3.821 | 1.497 |
| 2020-07-01 | 1.117 | 2.577 | 1.847 |
| 2020-10-01 | 1.179 | 2.446 | 1.813 |
| 2021-01-01 | 0.801 | 1.919 | 1.36 |
| 2021-04-01 | 0.401 | 1.11 | 0.755 |
| 2021-07-01 | 0.362 | 0.916 | 0.639 |
| 2021-10-01 | 0.328 | 0.827 | 0.578 |
| 2022-01-01 | 0.293 | 0.816 | 0.555 |
| 2022-04-01 | -0.343 | 0.838 | 0.248 |
| 2022-07-01 | 0.292 | 0.789 | 0.541 |
| 2022-10-01 | 0.322 | 0.813 | 0.568 |
| 2022-12-01 | 0.333 | 0.81 | 0.572 |

*Sources*: Ministry of Health, IBGE, TSE; authors’ calculations.

However, we are facing a trade-off between: trying to control at least partially the bias associated with unobservables or tackling the substantial overdispersion of the data on mortality. When overdispersion is present in the data, fitting a standard Poisson regression model may lead to incorrect inference and predictions. We have therefore decided to present also the results of the Poisson regression model with a fixed effect in the main tables (tables 2 and 4) in the revised version of the manuscript, but the following stages of the analysis focus on the results of the Negative Binomial model.

Brown, C.S., Ravallion, M. (2020), “Inequality and the coronavirus: Socioeconomic covariates of behavioral responses and viral outcomes across US counties”. *NBER Working Paper* 27549, July.

Caitlin S. Brown & Martin Ravallion, 2022. "[Inequality and Social Distancing during the Pandemic](https://ideas.repec.org/p/nbr/nberwo/30540.html)," [*NBER Working Papers*](https://ideas.repec.org/s/nbr/nberwo.html) 30540, National Bureau of Economic Research, Inc.

Tables should offer comprehensive details regarding variable measurements and technical information, reducing the need for readers to refer back to the article for clarification.

We took this suggestion into account. Tables have been modified to facilitate the reading. We made explicit some technical information on variables measurement and more detailed information are given in the appendix.

Furthermore, I recommend excluding the last columns of Tables 1 and 2 if you do not intend to interpret the controls. This can streamline the presentation of results and emphasize the key findings.

We took this suggestion into account. we excluded the last column in Tables 2 and 4 (as meant by the reviewer). As already specified, we substituted the last column by our new estimations based on Poisson regression with fixed effects.

For a scientific journal, it is advisable to consider shortening your article to enhance clarity and improve communication with your readers.

We took this suggestion into account. We tried to reduce certain parts of the manuscript to make them clearer.

Lastly, given the causal nature of your research, it is imperative to clarify the causal interpretation of the main coefficient and provide robust justification. Explain why this coefficient can be interpreted causally and offer supporting evidence or reasoning.

In the introduction, and especially in section 3, we explained in detail the mechanisms and arguments that support our hypotheses on the effect of Bolsonaro's position on the mortality rate (an effect that involves, in particular, compliance with social distancing and vaccination) in the municipalities where his most fervent supporters are concentrated.

Besides, we also made clear that the nature of the phenomenon analysed, with observational data, does not allow a causal relationship to be rigorously identified. However, our specifications, which consider a large number of controls (municipality characteristics), highlight significant and informative conditional correlations between political factors and the Covid-19 mortality rate.

Nevertheless, in order to take better account of this remark, we introduced some comments on this issue in the conclusion (see also the answer to Reviewer#1 last comment). In general, to estimate a rigorous causal impact, two main empirical strategies can be implemented: IV estimates or RCTs. In the first case, we (and the literature) have not identified any credible instrument. As for the second, our study is based on observational data and not on an experimental data. More generally, there is no way of identifying the "Bolsonaro effect" in such an experimental framework. This is a limitation of our paper, already mentioned in the submitted version, which we have further emphasised in the revised manuscript.
