# Supplementary material for: Investigating the ‘Bolsonaro effect’ on the spread of the Covid-19 pandemic: An empirical analysis of observational data in Brazil
Source: PLoS One. 2024 Apr 18;19(4):e0288894. doi: 10.1371/journal.pone.0288894 (PMC11025779; doi:10.1371/journal.pone.0288894)
Supplement: Attachment — Submitted filename: ReviewPLOSONE2024.odt [file pone.0288894.s012.odt]

Review “Investigating the 'Bolsonaro effect' on the spread of the Covid-19 pandemic” - 
PLOS ONE

Thank you for giving me the opportunity to review the revised version of “Investigating the 'Bolsonaro effect' on the spread of the Covid-19 pandemic.”

I find the article promising, but it still requires several improvements before it can be published.

Firstly, I noticed that the revised version is missing the figures and the appendix. A letter from the authors explaining the revisions would have been highly beneficial for me.

On page 12, the claim that “a significant effect at the municipal level guarantees significance in terms of individual probabilities” is not accurate. This overlooks the ecological fallacy, an important statistical concept. Significant effects at a municipal level do not necessarily translate to similar relationships at an individual level, as aggregate data can mask significant variations. Thus, it's crucial to analyze and interpret data at the appropriate level to avoid incorrect inferences.

Still on page 12, the authors’ dismissal of the potential influence of omitted variables correlated with political factors is unconvincing. Many factors could impact the relationship between support for Bolsonaro and the spread of Covid-19. For example, pre-existing characteristics of the municipalities, such as distrust in science or social behavior patterns, might influence this relationship. I believe the authors should focus on acknowledging the limitations of their research design rather than defending it weakly.

I suggest that on page 13, instead of using bullet points, a chronological figure would be more effective for clarity.

The authors do not disclose the controls in their regressions, which makes the analysis on pages 18 and 23 challenging to interpret. They should spend more time explaining their choice of controls, referencing the work of Carlos Cinelli, Andrew Forney, and Judea Pearl from 2022 on differentiating between “good” and “bad” controls.

Since the authors frequently praise Brazil’s immunization system, I believe it is essential for them to present more substantial evidence and cite relevant literature to support these claims.

For minor comments:
- In the first paragraph, I suggest using an active voice and clearly stating that Brazil had the second-highest absolute number of Covid-19 deaths.
- The term 'Covid-19' should be used consistently throughout the text.
