# Supplementary material for: Investigating the ‘Bolsonaro effect’ on the spread of the Covid-19 pandemic: An empirical analysis of observational data in Brazil
Source: PLoS One. 2024 Apr 18;19(4):e0288894. doi: 10.1371/journal.pone.0288894 (PMC11025779; doi:10.1371/journal.pone.0288894)
Supplement: Attachment — Submitted filename: Response to Reviewers -Investigating Bolsonaro effect-Rev2.docx [file pone.0288894.s013.docx]

**Investigating the ‘*Bolsonaro effect*’ on the spread of the Covid-19 pandemic:**

**an empirical analysis of observational data in Brazil**

**Detailed answers to Reviewers**

We would like to thank the editor and reviewers for their positive assessment of our article and their valuable comments. The suggestions helped us to improve the quality of the document. We have taken them all into consideration in our revision. We believe that the manuscript has been significantly improved and we hope that this revised version meets the journal's requirements for publication. In any case, we remain at your disposal if you have any further questions.

Please find below our responses (in black) regarding the points you raised (in blue). To facilitate the identification of our modifications, they appear in the revised manuscript (revision 2) as ‘track changes’.

Reviewer #1: The article has been carefully revised, taking into account the comments of the two reviews. The revision has helped improve the article and the result is now suitable for publication.

We would like to thank reviewer #1 for this comment in recognition of the efforts made to carefully revise and improve the manuscript (responding to and considering all the comments made in the previous review).

Reviewer #2: Thank you for giving me the opportunity to review the revised version of “Investigating the 'Bolsonaro effect' on the spread of the Covid-19 pandemic.”

Thank you for giving me the opportunity to review the revised version of “Investigating the 'Bolsonaro effect' on the spread of the Covid-19 pandemic.”

I find the article promising, but it still requires several improvements before it can be published.

Firstly, I noticed that the revised version is missing the figures and the appendix. A letter from the authors explaining the revisions would have been highly beneficial for me.

First, we must stress that we have prepared and sent an extensive Answer to the Reviewers, with special attention to Reviewer#2 comments, who carefully reviewed our paper in the previous round (revision 1). We deeply regret the fact that reviewer 2 did not receive our detailed responses which consider all the comments (as pointed out by reviewer#1; see above).

The figures and the appendix were also sent with the revised manuscript and the response to the reviewers.

In his new review by Reviewer#2, some comments were already addressed in our first “Answer to the Reviewers”, while other are new comments, not stressed earlier. Thus, we will send our initial (and augmented) answers to the old comments (taking advantage to re-send the full Answer) and elaborate on the new queries.

Reviewer #2:

On page 12, the claim that “a significant effect at the municipal level guarantees significance in terms of individual probabilities” is not accurate. This overlooks the ecological fallacy, an important statistical concept. Significant effects at a municipal level do not necessarily translate to similar relationships at an individual level, as aggregate data can mask significant variations. Thus, it's crucial to analyze and interpret data at the appropriate level to avoid incorrect inferences.

First, we make it clear in the paper that we do not interpret our results in terms of individual probabilities. Our approach and the analysis is carried out at the municipal level.

Regarding the comment on the inaccuracy of this specific sentence “a significant effect at the municipal level guarantees significance in terms of individual probabilities”, we disagree with this point. Let us suppose there is no individual effect (correlation, conditional or unconditional) at the individual level (take for instance by gender). Necessarily, there should not be any effect at the aggregate (here municipal) level (P => Q). In consequence, as (No Q => No P), an effect at the aggregate (municipal) level implies necessarily the existence of an effect at the individual level. What is not known is the sign of the effect, although the probability the correlation changes its sign is limited, but it is still possible.

This argument is explicitly developed in:

McLaren, J. (2021). Racial disparity in COVID-19 death: Seeking economic roots with Census data. *The B.E. Journal of Economic Analysis & Policy*, 21(3), Doi: [10.1515/bejeap-2020-0371](http://dx.doi.org/10.1515/bejeap-2020-0371), p.899:

‘*The data used are US county aggregates of COVID-19 mortality over time, matched with socioeconomic and demographic data by country. The mortality data are not broken down by race, but in principle one can still test for and measure racial disparity by looking for correlation between the size of a county’s minority population share and the county’s mortality rate. If there were no difference in mortality between a minority group and the rest of the population, the size of the minority share would have no effect on the county’s mortality rate, so this correlation provides indirect evidence of a disparity in mortality rates. The exercise follows two steps: first, confirm the existence of a racial disparity by using a regression to show that minority population shares are correlated with mortality rates. Second, control for a range of county-level socio-economic factors to see if the racial disparity weakens or disappears. If it does, then that stands as evidence that the socioeconomic factor is part of the reason for the racial mortality discrepancy*”

In our paper, we have specified that :

“*However, this type of approach has its limitations and the results should be interpreted with due caution. First, analysis by municipalities cannot be interpreted in terms of individual risks. However, we know that a significant effect at municipal level also guarantees significance in terms of individual probabilities.^[1]^ We can assume, then, that the individual and municipal approaches generally converge in terms of signs (McLaren, 2021). Otherwise, inverse mechanisms would need to be explained.^[2]^*”

Reviewer #2:

Still on page 12, the authors’ dismissal of the potential influence of omitted variables correlated with political factors is unconvincing. Many factors could impact the relationship between support for Bolsonaro and the spread of Covid-19. For example, pre-existing characteristics of the municipalities, such as distrust in science or social behavior patterns, might influence this relationship. I believe the authors should focus on acknowledging the limitations of their research design rather than defending it weakly.

New comment. The reviewer is right: the omitted variable bias cannot be excluded. But this is the case of all analysis on observational data (unless credible empirical strategies – like IV or RDD – can be implemented; which is not our case). However, in the specific example of political factors mentioned by the reviewer (distrust in science, social behaviour patterns), we do test this potential bias, at least for vaccination. As the “Bolsonaro effect” is not significant on other vaccines in 2019 (Bolsonaro was not in charge; placebo test), but only since 2020 on Covid-19 vaccination (and other vaccines), we can plausibly induce that we are capturing a real “Bolsonaro effect”, based on his specific attitudes and behaviours regarding the pandemic. Furthermore, logistic regressions based on an individual survey sponsored by Sou Ciência in 2022 show the “Bolsonaro effect” holds controlling for religion (evangelic, correlated with Bolsonaro support and anti-science attitudes) and other sociodemographic variables on vaccination, Covid-19 testing and masks (results available upon request to the authors).

In all case, we explicitly highlight the potential issue of omitted variables (see our first Answer to the Reviewers).

In our paper, we have specified that :

“*the econometric models tested here can be used to estimate the correlations between the mortality rate and different factors, corrected for structural effects. However, as in most analyses of observational data, we identify correlations that do not necessarily imply causality. For example, it is quite plausible that restrictions were applied more strictly in municipalities where mortality rates were already higher. It is therefore hard to disentangle the actual impact of the restrictive measures. Finally, there is the issue of potentially omitted variables correlated with political factors. In this case, the correlation observed in our model might be influenced by these omitted variables. Yet it is hard to find convincing arguments to support this reservation. Considering the data on comorbidities most often cited as examples of omitted variables, it is not clear to what extent they might be correlated with political factors*” (Section 3.1, Empirical approach);

and “*Although the nature of the phenomenon analysed, with observational data, does not allow a causal relationship to be clearly identified, our specifications, which consider a large number of characteristics of municipalities, highlight significant and informative conditional correlations between political factors and the Covid-19 mortality rate*.” (Conclusion)

Reviewer #2:

I suggest that on page 13, instead of using bullet points, a chronological figure would be more effective for clarity.

We thank the reviewer for this suggestion, which makes the presentation clearer.

A figure presents the timeline of the Covid-19 pandemic instead of bullet points.

Reviewer #2:

The authors do not disclose the controls in their regressions, which makes the analysis on pages 18 and 23 challenging to interpret. They should spend more time explaining their choice of controls, referencing the work of Carlos Cinelli, Andrew Forney, and Judea Pearl from 2022 on differentiating between “good” and “bad” controls.

First, we would like to point out that the complete tables with the controls are available in the appendix provided with the manuscript (table S2, S3, S4, etc.). We have been advised to simplify the presentation of the tables in the body of the text, bearing in mind that our main objective is to better identify the 'Bolsonaro effect' rather than to interpret all the controls per se.

But, we would like also to thank the reviewer for this suggestion to provide more explanation on the choice of control variables. We agree, and we think that quoting this reference (Cinelli et al., 2022) is highly relevant for identifying bad and good controls. We have therefore taken into consideration this suggestion.

However, as we have already been asked to shorten the manuscript, we have essentially added explanations in cases where the choice of introducing controls was debatable and potentially problematic.

In reference to Cinelli et al. (2022), we consider in our models a broad spectrum of controls: 1/variables with a potential direct or indirect effect on the mortality rate (neutral control, possibly good for precision) and above all, 2/ confounding variables which potentially might have influence on the mortality rate and the political factor (good controls aiming at blocking back-door paths).

We specified in the manuscript that “Among the control variables, the vaccination rate and mobility indicator are two transmission factors that could be considered. However, their inclusion in the model would potentially absorb at least part of the effect of our variable of interest (the political factor). This could lead to overcontrol, as our interest is in the total effect (Cinelli et al., [2022](https://link.springer.com/article/10.1007/s11205-023-03118-5#ref-CR12)). In addition, it should be noted that the introduction of these two variables could engender a reverse causality problem in model 1. … In the case of the vaccination rate, when considering the 2nd dose of the vaccine, controlling for the 1st dose is a way to purge the reverse causality effect from the ratio of mortality to the number of vaccines. So, the vaccination rate remains in one of our specifications, but since it could be classified as a ‘bad control’ or a collider, our preferred specification also excludes the vaccination rate.”

In the discussion, we acknowledge that the introduction of the vaccination rates as control variables could be questioned [Cinelli et al., [2022](https://link.springer.com/article/10.1007/s11205-023-03118-5#ref-CR12)], but, we have decided to keep this step as a sensitivity test.

Reviewer #2:

Since the authors frequently praise Brazil’s immunization system, I believe it is essential for them to present more substantial evidence and cite relevant literature to support these claims.

We thank the reviewer for this recommendation.

Even though we have already cited references highlighting the success of the vaccination programme in Brazil:

*46. Bernardeau-Serra, L., Nguyen-Huynh, A., Sponagel, L., Sernizon Guimarães, N., Teixeira de Aguiar, R.A., Soriano Marcolino, M. (2021), “The COVID-19 Vaccination Strategy in Brazil – A Case Study”, Epidemiology 202 (2): 338-359.*

*60. Larson, H.J., de Figueiredo, A., Xiahong, Z., Schulz, W.S., Verger, P., Johnston, I.G., Cook, A.R., Jones N.S. (2016), “The State of Vaccine Confidence 2016: Global Insights Through a 67-Country Survey”, EbioMedicine, 12: 295-301. doi: 10.1016/j.ebiom.2016.08.042.*

We provide here additional references which support the assertion that the National Immunization Program (NIP) is considered as one of the most successful immunization programs. We have included two of them in our revised manuscript.

*Homma, A., Martins, R. d. M., Leal, M. d. L. F., Freire, M. d. S. & Couto, A. R. (2011), ‘Atualização em vacinas, imunizações e inovação tecnológica’, Ciência e Saúde Coletiva 16(2), 445–458.*

In this study, researchers from Fiocruz claim that:

“The NIP is the most effective programme among emerging countries and is comparable to that of developed countries. Its enormous growth can be demonstrated with the following figures: in 2000, the budget was R$200 million; it rose to R$825 million in 2009, offering 26 different types of vaccines....”

*Domingues, C. M. A. S., Maranhão, A. G. K., Teixeira, A. M., Fantinato, F. F. S., & Domingues, R. A. S. (2020). 46 anos do Programa Nacional de Imunizações: uma história repleta de conquistas e desafios a serem superados.* Cadernos De Saúde Pública*,* 36*, e00222919. <https://doi.org/10.1590/0102-311X00222919>*

*Sato, A. P. S.. (2018). What is the importance of vaccine hesitancy in the drop of vaccination coverage in Brazil?. Revista De Saúde Pública, 52, 96.* [*https://doi.org/10.11606/S1518-8787.2018052001199*](https://doi.org/10.11606/S1518-8787.2018052001199)

*Pércio J, Fernandes EG, Maciel EL, Lima NVT. 50 years of the Brazilian National Immunization Program and the Immunization Agenda 2030. Epidemiol Serv Saude. 2023 Dec 4;32(3):e20231009. doi: 10.1590/S2237-96222023000300001.*

**For minor comments:**

Reviewer #2:

- In the first paragraph, I suggest using an active voice and clearly stating that Brazil had the second-highest absolute number of Covid-19 deaths.

Many thanks, we took into consideration this suggestion and made the changes in the manuscript.

Reviewer #2:

- The term 'Covid-19' should be used consistently throughout the text.

We are sorry but we do not understand this comment. We had our paper proofread by a native English speaker. On checking, we used the term Covid-19 consistently to refer to the Coronavirus disease 2019. In the text, we use the expression "the Covid-19 pandemic" on a few rare occasions when we consider it necessary to emphasise the fact that this is a pandemic.

1. If the mortality rate is different between two population categories (e.g. Poor and Non-Poor) at aggregate level, it should also be different at individual level (likewise, if the mortality/contamination rate is the same at individual level, it should also be the same at aggregate level).
2. For example, if the municipalities with higher male ratios are harder hit, it does not necessarily mean that males are hardest hit. Women in municipalities where male ratios are high could be most at risk of infection. However, this would imply finding a particular mechanism to support this view.
